# Inter-fractional monitoring of $^{12}$C ions treatments: results from a clinical trial at the CNAO facility

**DOI:** 10.1038/s41598-020-77843-z

**Published:** 2020-11-26

**Authors:** M. Fischetti, G. Baroni, G. Battistoni, G. Bisogni, P. Cerello, M. Ciocca, P. De Maria, M. De Simoni, B. Di Lullo, M. Donetti, Y. Dong, A. Embriaco, V. Ferrero, E. Fiorina, G. Franciosini, F. Galante, A. Kraan, C. Luongo, M. Magi, C. Mancini-Terracciano, M. Marafini, E. Malekzadeh, I. Mattei, E. Mazzoni, R. Mirabelli, A. Mirandola, M. Morrocchi, S. Muraro, V. Patera, F. Pennazio, A. Schiavi, A. Sciubba, E. Solfaroli Camillocci, G. Sportelli, S. Tampellini, M. Toppi, G. Traini, S. M. Valle, B. Vischioni, V. Vitolo, A. Sarti

**Affiliations:** 1 Dipartimento di Scienze di Base e Applicate per l’Ingegneria, Sapienza Università di Roma, Rome, Italy; 2 Dipartimento di Fisica, Sapienza Università di Roma, Rome, Italy; 3 INFN Sezione di Roma I, Rome, Italy; 4 INFN Sezione dei Laboratori di Frascati, Rome, Italy; 5 Museo Storico della Fisica e Centro Studi e Ricerche “E. Fermi”, Rome, Italy; 6 INFN Sezione di Milano, Milan, Italy; 7 INFN Sezione di Pisa, Pisa, Italy; 8 Dipartimento di Fisica, Università degli Studi di Milano, Milan, Italy; 9 Dipartimento di Fisica “E. Fermi”, Università di Pisa, Pisa, Italy; 10 INFN Sezione di Torino, Turin, Italy; 11 CNAO Centro Nazionale di Adroterapia Oncologica, Pavia, Italy; 12 Scuola di Specializzazione in Fisica Medica, Sapienza Università di Roma, Rome, Italy; 13 INFN Sezione di Pavia, Pavia, Italy; 14 Dipartimento di Chimica e Chimica Industriale, Università di Pisa, Pisa, Italy; 15 Dipartimento di Elettronica Informazione e Bioingegneria, Politecnico di Milano, Milan, Italy; 16 Scuola di Specializzazione di Fisica Medica, Sapienza Università di Roma, Rome, Italy

**Keywords:** Radiotherapy, Experimental nuclear physics

## Abstract

The high dose conformity and healthy tissue sparing achievable in Particle Therapy when using C ions calls for safety factors in treatment planning, to prevent the tumor under-dosage related to the possible occurrence of inter-fractional morphological changes during a treatment. This limitation could be overcome by a range monitor, still missing in clinical routine, capable of providing on-line feedback. The Dose Profiler (DP) is a detector developed within the INnovative Solution for In-beam Dosimetry in hadronthErapy (INSIDE) collaboration for the monitoring of carbon ion treatments at the CNAO facility (Centro Nazionale di Adroterapia Oncologica) exploiting the detection of charged secondary fragments that escape from the patient. The DP capability to detect inter-fractional changes is demonstrated by comparing the obtained fragment emission maps in different fractions of the treatments enrolled in the first ever clinical trial of such a monitoring system, performed at CNAO. The case of a CNAO patient that underwent a significant morphological change is presented in detail, focusing on the implications that can be drawn for the achievable inter-fractional monitoring DP sensitivity in real clinical conditions. The results have been cross-checked against a simulation study.

In Particle Therapy (PT) solid tumors are treated using charged ion beams, mainly protons and $^{12}$C ions. In the case of carbon ions, due to the enhanced Relative Biological Effectiveness and the lower Oxygen Enhancement Ratio with respect to the conventional radiotherapy, a high precision in the dose released to the target volume is combined with a greater efficacy in damaging the cancerous cells^1^. Furthermore, there is also a renewed interest in using other ions, like He or O, exploring their different radiobiological characteristics to implement clinical centres capable of delivering optimized, tumor- and patient-specific, treatments^2–5^.

The intrinsic high dose conformity achievable in PT currently requires the implementation of safety factors (up to 2–3% on the total range^6^) in treatment planning to account for the beam range uncertainties, typically due to Computed Tomography (CT) scan mis-calibration, uncertainties in the Hounsfield Units—stopping power conversion, as well as inter-fraction morphological changes or uncertainty on patient positioning. Nowadays, an in-vivo technique capable of measuring with the required precision the actual dose released in the patient is missing in clinical routine. Safety margins are hence used when defining the Planned Treatment Volume to guarantee the medical prescription avoiding target underdosage^7,8^.

A carbon ion therapy treatment can last several weeks: a CT scan is performed before the treatment as input for the Treatment Planning Software (TPS) and, for some selected pathologies, after the delivery of the first ten fractions. Within such time window the patient morphology may change dramatically: tumor regression occurs when the therapy is effective and the insurgence of beam-induced internal toxicities is frequent for different kinds of pathologies. Such inter-fraction morphological changes may alter the dose deposition in the patient with respect to what was planned by the TPS.

In the current clinical practice, a re-evaluation CT is done only when major morphological variations are expected and it is planned after the delivery of a number of fractions pre-defined on the basis of the physician’s experience. In all the other cases no CT is foreseen, to avoid the additional (and, in most cases, unnecessary) dose release to the patients. Whenever the re-evaluation CT information identifies the insurgence of a morphological variation that significantly affects the dose release in the tumor region, the treatment is re-planned. Such strategy has two clear shortcomings: there is a fraction of patients in which the change is expected but it does not happen (and in this case the additional dose from the imaging is unnecessary) and, at the same time, some patients can develop significant morphological changes even if the clinical statistics acquired so far refers that event as unlikely.

An experimental technique capable of monitoring the morphological changes and identifying when they significantly alter the dose deposition pattern inside the patient is eagerly needed as it would finally allow to perform the re-evaluation imaging only when really necessary. A possible way of monitoring the dose deposition inside the patient is to exploit the secondary particles produced during the treatment.

Several beam range monitor techniques exploiting the detection of secondary particles have been proposed in the last decades: prompt-gamma based detectors^9–11^, PET-like detectors that use the beam-induced activation of $\beta ^+$ emitters^12–15^ and charged fragments detectors^16^.

In a carbon ion treatment, protons and neutrons are the most abundant products of the beam fragmentation inside the patient^17^. A significant fraction of the protons produced at large angles with respect to the beam direction has enough kinetic energy to escape from the patient, as reported in several measurements^18–21^. The production yield of the fragments along the beam path depends on the density and on the atomic mass of the crossed tissues. Beam range variations can be therefore identified, during the treatment, exploiting the distribution of the charged fragments production points. An approach based on the detection of charged fragments suffers from two main shortcomings: the particles need a kinetic energy of the order of 50–100 MeV to escape from the patient and suffer from multiple scattering (MS) interactions. The MS introduces an uncertainty on the estimate of the fragments emission position of the order of 5–10 mm, depending on the treatment topology (tumor depth, direction of the treatment fields) and fragment production point. Moreover, fragments undergo a different absorption depending on the travelled path inside the patient, making the correlation between the beam range and the reconstructed emission profile non-trivial. However, unlike other techniques based on the detection of photons (either prompt or generated by $\beta ^+$ emitters), the charged particles detection can be performed with high efficiency, as the fragments escaping the patient are mainly low energy protons, in an almost background-free environment (the only significant source of protons is represented by the beam fragmentation).

The Dose Profiler (DP)^22^ is a charged particle tracker designed to operate as a carbon ions beam range monitor at the CNAO (Centro Nazionale per l’Adroterapia Oncologica, Pavia, Italy) therapy center. The DP is able to backtrack the fragments and to measure the emission point of the charged particles produced during a treatment^23^.

The DP has been developed within the framework of the INSIDE (INnovative Solution for In-beam Dosimetry in hadronthErapy) project^24^ with the goal of implementing and testing the first simultaneous bi-modal system ever built for the detection of charged fragments and $\beta ^+$ emitters that can be used both for carbon and proton treatments online monitoring.

Exploiting this device we propose a method, based on the comparison of the reconstructed emission position distribution of the fragments collected during each treatment session, to identify the inter-fractional morphological changes. The time scale for the appearance of significant morphological variations is of the order of days. In this paper we propose a method capable of providing to the physician and clinicians a feedback on the treatment quality, as well as a reliable criterium for rescheduling the treatment plan performing a new CT scan.

The DP dis-homogeneities spotting capability has been studied in a clinical environment using the data collected during the INSIDE clinical trial^25^ that is being carried out at CNAO. The data collected from the first patients ever monitored online during carbon ions treatments will be presented and the results discussed in the context of inter-fractional monitoring sensitivity. The obtained results have also been cross-checked against a detailed Monte Carlo (MC) simulation implemented with the FLUKA^26,27^ MC code using the CT scans of a patient affected by Adenoid Cystic Carcinoma (ACC), treated at CNAO.

The paper is organized as follows. “Materials and methods” describes how the treatment fractions can be monitored using charged particles and the data and MC samples used in the analysis. “Results” describes the obtained results, related to the first data collected during the clinical trial and to the relative MC study. “Discussion” presents a discussion of the obtained results in the context of carbon ion radiotherapy treatments monitoring.

## Materials and methods

The morphological changes identification capability of the DP has been assessed analysing the data collected monitoring the treatment of patients affected by ACC. A single patient was selected, among the cohort of the first ten monitored patients, as the re-evaluation CT scan showed that a significant density change in the tissues crossed by the beam had occurred. We used this case to benchmark the technique capabilities in a case where a significant variation occurred.

The results were validated using a MC simulation. The CT scans of the patient, performed for the treatment planning and after the delivery of the first eight fractions, were imported and used in a full simulation performed with the FLUKA MC software. The comparison of the results obtained using the two CTs was used to cross check the data analysis.

In the following we shortly review the DP main characteristics and present the details about how the reconstruction and the simulation of the events were configured and carried out, using the information from the patient CT scans and the $^{12}$C beams characteristics (fluency, direction and energy) taken directly from the Treatment Plan (TP). Finally, a description of the technique adopted to spot the inter-fraction morphological changes will be given.

### The Dose Profiler

**Figure 1:**
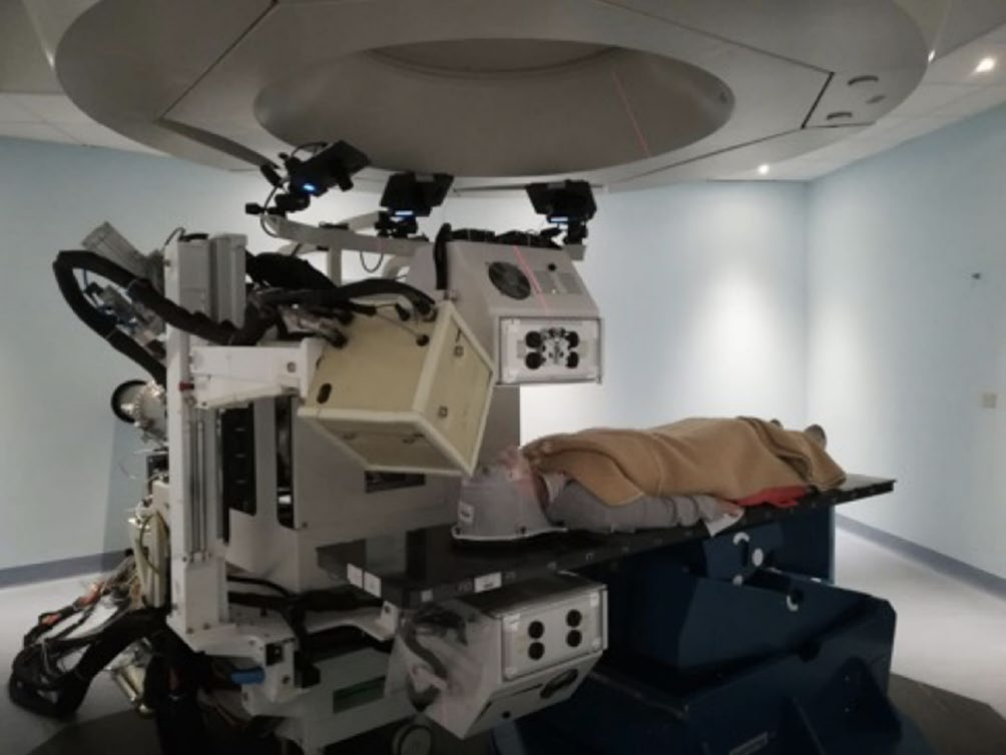
View of the INSIDE cart with the DP (beige box) and the PET detectors (white boxes above and below the patient bed) installed in the CNAO treatment room n.1, which has a fixed horizontal beam line. The image shows a view of the first patient treated with $^{12}$C ions that has been monitored by means of the DP.

The DP^22,23^ is a compact detector with an overall size of $\sim$ 30 $\times$ 30 $\times$ 30 cm$^3$ composed of orthogonal layers of scintillating fibers read-out by Silicon PhotoMultipliers (SiPMs). Charged fragments that pass through the detector producing a light signal can be measured with very high efficiency ($\sim$ 90% per layer) in a nearly background free environment. The fragment emission point resolution, measured back-tracking towards the treatment room iso-center from the DP nominal position, is about 7 mm for each particle, a value is compatible with the expected average multiple scattering undergone by the fragments in their exit path from the patient body^18^. An optimized DAQ system provides a dead time per event of $\sim$ 5 $\upmu$s, needed to sustain the typical fragment expected rate (that can be as large as *O*(100) kHz). Since the CNAO accelerator instantaneous intensity, varies with time, the dead time correction plays a crucial role when comparing the data acquired in different fractions of the same treatment. The procedure followed to account for such effect when looking for inter-fractional morphological changes is explained in § "Results".

The DP is integrated in the INSIDE movable cart that includes also two planar PET heads^13,14^ and is placed at $\sim$ 50 cm from the treatment room isocenter due to the constraints set by the mechanical integration with the PET device. The DP position inside the CNAO treatment room n.1 can be seen in Fig. 1 that shows the first patient treated with $^{12}$C ions that has been monitored. In the operational position, during the treatment monitoring, the DP is tilted at 60$^\circ$ with respect to the beam direction, and 30$^\circ$ upwards.

The DP position is fixed in the INSIDE cart, which is locked in proximity of the beam nozzle when operating and has to be placed and removed from the measurement position in each fraction. The uncertainty relative to the positioning of the detector ($\sim$ 1–1.5 mm along the x,y and z axis) has been quantified by means of a survey done using a precise laser tracker and has been properly accounted for when assessing the technique performance in comparing different fractions (for more details see § "Results").

### The clinical trial

The results presented hereafter refer to the analysis of the data collected monitoring three patients enrolled in a clinical trial^25^ carried out at CNAO in treatment room n.1. At CNAO, a typical treatment with carbon ions lasts about 4 weeks. The details about the treatment plans of the patients are listed in Table 1. For each patient we list: the number of fractions in which the treatment plan is divided (for PZA and PZC the total number of fractions for each of the two different irradiation configurations is shown); if a re-evaluation CT was performed, how many fractions passed since the treatment start; the angles for each field in a given fraction; the total treatment dose. The angle at which the field is delivered is computed in the treatment room reference frame: 0$^\circ$ and 270$^\circ$ correspond to the position in which the patient bed is placed as shown in Fig. 1, and the position in which the patient body is aligned with the beam direction, respectively.

In this manuscript we present the data relative to three patients treated for ACC of minor salivary glands of the head and neck district. The clinical study was performed in accordance with all the relevant guidelines and running regulations on clinical trials and was approved by the referral ethics committee “CNAO” with the code CNAO-OSSINSIDE-02-18 on july 31, 2019; the informed consent was obtained from all the adult participants enrolled. No information or images that could lead to identification of the participant are present in this work.

Out of all the monitored patients only patient PZC showed some morphological changes, but none of them was significant enough to justify a re-planning. The clinical routine defines how and when the measurements are performed and has an impact also on the interpretation of the results. As a first step, the patient is positioned accordingly to the prescriptions of the treatment planning system. Once the correct positioning of the patient is verified using X-rays, the INSIDE cart is moved into the measuring position and the DP is placed in the data taking position. If, when moving from a given field to the other one, the position of the patient needs to be re-checked using the X-rays, the DP needs to be removed and put back to avoid any interference with the X-ray tubes. All these operations have no impact on the patient positioning, as the detectors are fixed to the beam line and not interfere in any way with the patient couch, and add only few seconds to the treatment setup. The cart positining is performed with a custom designed mechanical system that allows the insertion and removal of the apparatus in less than 30 s.

**Table 1:** Treatment plan information for patients included in the INSIDE clinical trial presented in this manuscript.

Patient ID	PZA	PZB	PZC
n. Fractions	9+7	20	9+7
re-eval CT	After 7 fr.	No	After 8 fr.
Field0 (angle)	0$^\circ$	155$^\circ$	0$^\circ$
Field1 (angle)	180$^\circ$	270$^\circ$	310$^\circ$
Field2 (angle)	270$^\circ$	/	/
Dose (GyE)	65.6	60	68.3

For the treatment of shallow tumors a Range Shifter (RS) is used, a solid water 3 cm thick layer positioned between the beam exit window and the patient along the beam path.

### Monte Carlo simulation

To evaluate the expected performance in detecting the insurgence of morphological changes by means of comparing the secondary charged fragments production maps in two different fractions, a Monte Carlo simulation study has been performed. A patient affected by ACC that underwent a re-evaluation CT imaging session after the first eight fractions were delivered (PZC in Table 1) was identified as a good candidate for the technique performance evaluation crosscheck.

**Figure 2:**
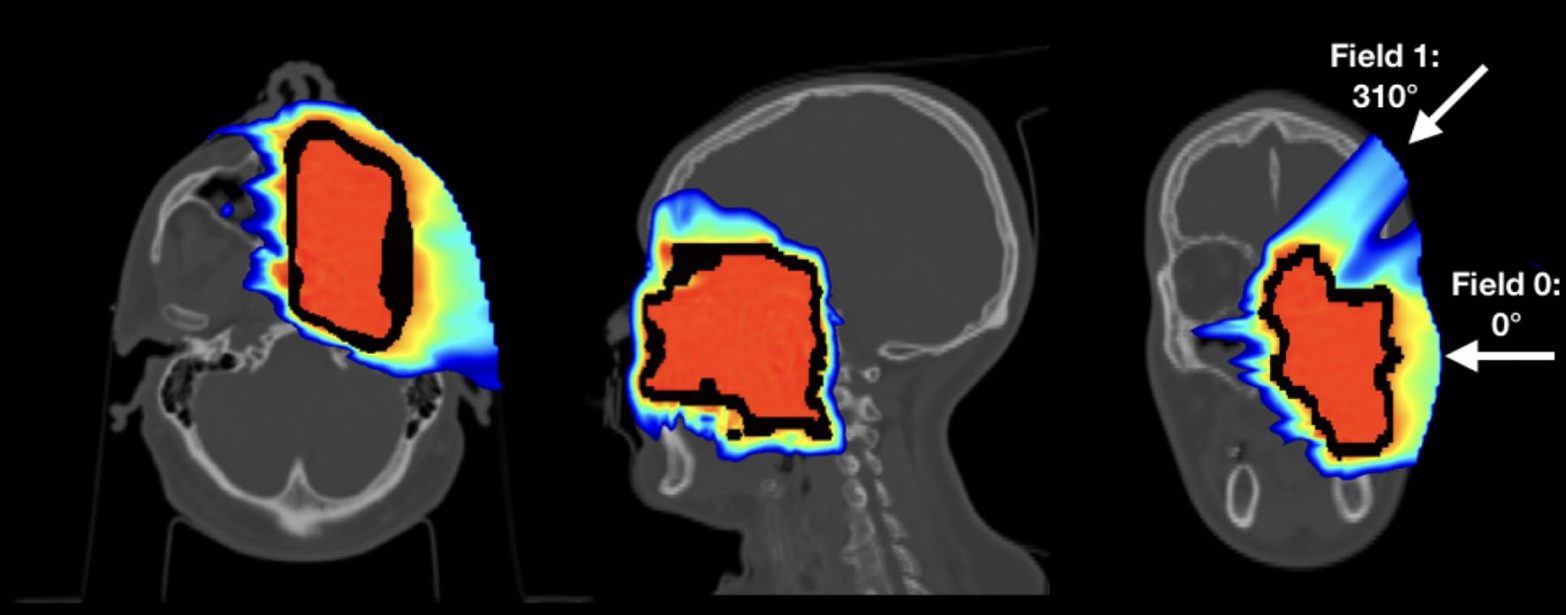
Treatment plan delivered to the patient selected for the MC study. In the horizontal plane the two different fields used to treat the patient can be seen. The black contour represents the planning Clinical Target Volume (CTV).

The original TP built accordingly to the first CT scan, whose dose distribution is shown in Fig. 2, foresaw two different fields (0$^\circ$, 310$^\circ$ in the CNAO reference frame). The RS has been included in both fields.

A different sensitivity to the morphological changes is expected depending on the incoming beam direction and tumor location, since secondary particles may cross different amount and types of tissues when escaping from the patient. The field at 310$^\circ$ has the most favourable topology for the fragment collection and has been chosen for our study.

The plan used for the simulation study consists of $\sim$ 33k Pencil Beams (PB), with kinetic energy in the range between 126 MeV/u and 278 MeV/u (57 slices), and a number of ions shot in each PB in the range between $\sim$ 10$^3$ and $\sim$ 7 $\times$ 10$^5$. The CTV volume is $\sim$ 5 $\times$ 10 $\times$ 10 cm$^3$. The FLUKA MC code was used to simulate the interactions of all the primary ions foreseen in the TP The beam nozzle and the RS are included in the setup accordingly to the measurements performed in the CNAO treatment room. The full DP mechanical and read-out details are implemented in the Monte Carlo simulation. The optical cross-talk between the fibers, the energy resolution and the layer detection efficiency are also taken into account to reproduce the experimental conditions^23^.

**Figure 3:**
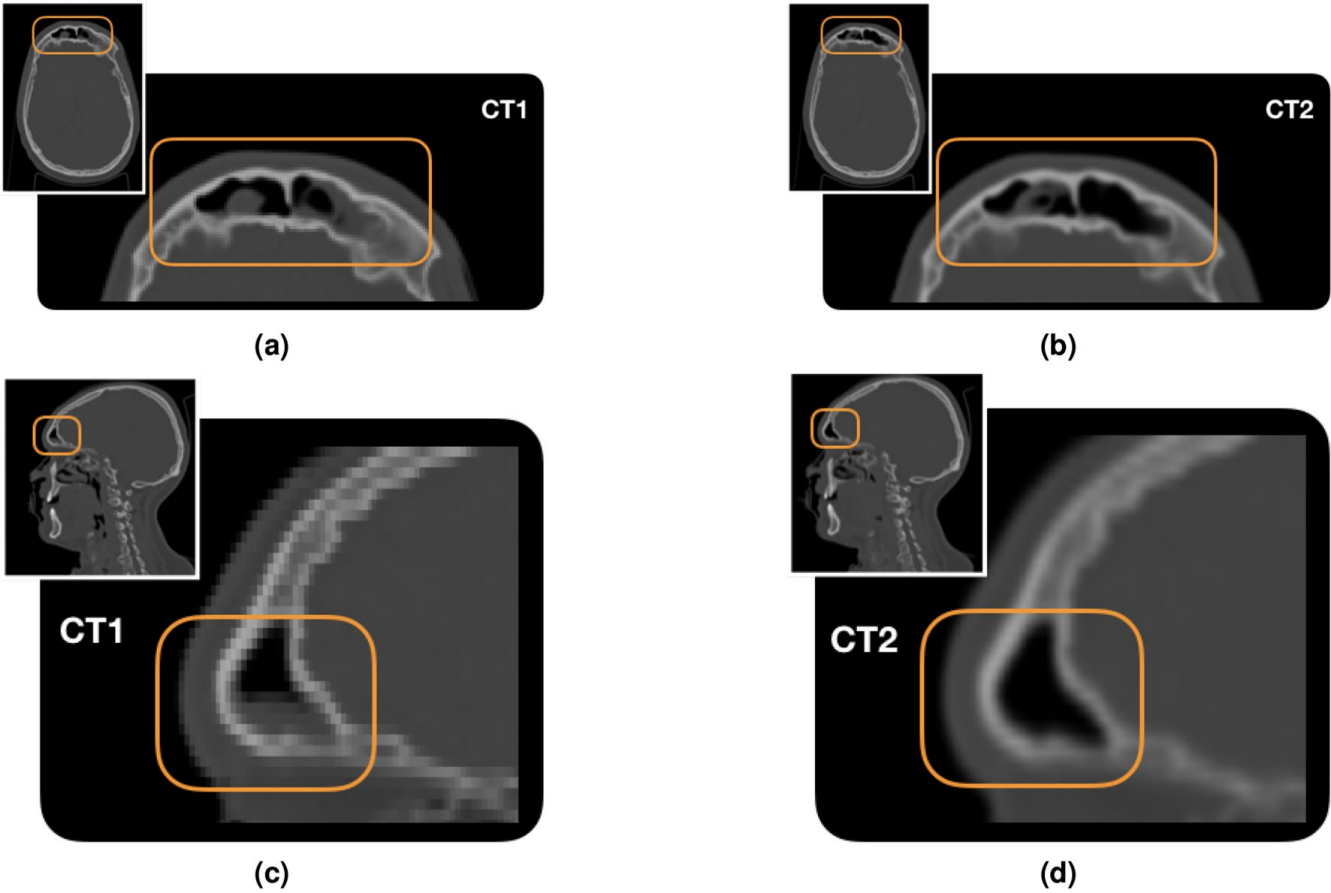
View of CT1 (left) and CT2 (right). The emptying of the nasal cavities induced by the treatment is highlighted with orange boxes.

To perform the study on the morphological changes, the two PZC CT scans were used for the simulation: the first one was acquired before the treatment and it was used for the treatment planning, while the second one was taken two weeks after the treatment start. We will refer to the simulation and control CT in the following as CT1 and CT2 respectively. In Fig. 3 the two CT scans in the ITK-SNAP^28^ user interface are shown. The regions highlighted in orange show the differences in the nasal cavities between the two CTs.

The same treatment plan delivery has been simulated using both CT scans, in order to compare the secondary fragments emission distributions.

The alignment of the CT images has been performed using the Insight Toolkit co-registration software tools^29,30^. The obtained level of agreement was compatible with the one obtained using the RayStation^31^ software applied for treatments optimisation at CNAO.

The interaction of all the carbon ions that are foreseen in a given fraction and field of the patient TP is followed in all the defined regions of the setup and the detector simulation is performed accordingly to previously published studies^23^. The track reconstruction and the fragment backtracking are performed in the very same way as in the real data reconstruction. The production points of the fragments are hence obtained as Points of Closest Approach (PCA) of the reconstructed track with the nominal incoming beam direction (as defined from the TP definition). The PCA 3D maps obtained from the simulated events are used as input for the studies presented in the following.

### Inhomogeneities identification strategy

The production, and possible consecutive absorption, of secondary fragments is correlated to the density of the crossed materials. The capability of the technique to spot morphological changes occurring between different fractions has been verified using a PCA maps comparison algorithm. The 1D projections of the PCA distributions along the beam axis directions have been used to allow a robust statistical interpretation of the results. The data belonging to several nearby PBs have been packed, to reduce the impact of statistical fluctuations on the results, using a strategy detailed in the next paragraphs. In the following, the presented results are hence based on the reconstructed 1D emission spatial distributions, projected along the beam axis (*z* in the reference frame), for each PB (or group of PBs) delivered in the treatment.

When comparing two different distributions, the statistics available in each sample can play a significant role in determining the technique sensitivity. For this reason a study of the number of reconstructed tracks per PB (and hence the number of reconstructed PCA) has been performed.

The results presented hereafter, obtained by studying the reconstructed tracks in a MC simulation sample based on the CT1 scan, strongly depend on the tumor location and size and are shown just to give to the reader an idea of the order of magnitude of the available data sample when monitoring a treatment.

In the simulation, the average number of detected tracks per PB is found to be $\sim$ 100 and a significant fraction of them (30–50% range, depending on the treatment details) originated from the RS and it is hence useless for the morphological study. To obtain a significant statistics of reconstructed tracks for the comparison study, a strategy for merging the information relative to PBs that are targeting nearby voxels has been adopted, as already done in the context of prompt-$\gamma$ spectroscopy^10^.

The choice was to merge in a single ’Super PB’ (SPB in the following) the PBs that have the end range within the same 1 cm $\times$ 1 cm $\times$ 0.6 cm volume, for a maximum of 75 PBs (each PB is targeting a voxel that has a 2 $\times$ 2 $\times$ 2 mm$^3$ volume). In this case, the average number of tracks per SPB becomes $\sim$ 1000. The optimization of the integration volume against the beam incoming direction is still ongoing, as it generally depends on the tumor dimension and location, however few integration volumes have been tested and the results were found to be consistent.

To identify a relative alteration in the density of the voxel traversed by the beam or by the fragments in their exit path, the 1D projections of the reconstructed PCA of each SPB in a given fraction are compared. We used as consistency tests to assess the 1D projections agreement both the $\chi ^2$ and the Kolmogorov–Smirnov (KS) to evaluate the dependence on the binning choice and the sensitivity to the presence of shifted peaks in the distributions. Both tests have been performed for all the data sets analysed in the following, and consistent results have been obtained in all cases. We have hence decided to report in the following only the p-values of the $\chi ^2$ test.

The first aim of the study performed both on data and MC samples was to ensure that the statistical fluctuations and the systematic uncertainties related to the adopted technique are properly taken into account when processing the data. This is to avoid the identification of false positives when no real morphological or density change had occurred. For this reason, the tests were performed using as input the information from a patient, included in the clinical trial, for which the re-evaluation CT imaging confirmed that no significant morphological changes occurred during the first eight fractions of the treatment.

The impact of statistical fluctuations when comparing two different fractions has been evaluated by producing two MC simulation samples using the same CT scan and treatment plan but using different random seeds and comparing the resulting secondary particles profiles. The level of agreement between the measured distributions has been evaluated using the $\chi ^2$ test.

The obtained result, a flat p-value distribution between 0 and 1 for all the tests SPBs, is consistent with the hypothesis of distributions that agree within the statistical uncertainties. We can hence conclude that statistical fluctuations are not likely expected to trigger false positives when looking for significant morphological changes.

We also studied the effects related to the data taking conditions that can mimic a density change affecting the PCA distributions.

When analysing the data collected during the treatment of a patient a crucial effect that has to be accounted for is the detector dead time. While it is true that the uncertainty on the absolute number of delivered ions per PB is negligible when considering monitoring applications, the instantaneous beam intensity can change significantly, resulting into a variation of the dead time (DT) that affects the measured distribution.

Another important effect that can mimic a change in the measured distribution is the inter-fraction variation of the patient-DP relative position, due to the limited precision achieved in the positioning of the detector and/or of the patient in the treatment room isocenter.

This systematic uncertainty has been evaluated using the data collected during the clinical trial. The tracks originating from the RS have been used as input for a MC study in which the DP position along the x, y and z axis was shifted randomly according to the laser tracker characterisation study accuracy results. The resulting average shifts have been used to evaluate a systematic uncertainty to be applied when testing the distributions agreement. To minimize the impact of the patient inter-fraction misalignment when comparing the measured distributions, we decided to exclude the corresponding bins of the profiles leading edge from the $\chi ^2$ computation.

The efficacy of the implementation of both corrections has been tested against the data collected in the clinical trial. Patients in which the re-evaluation CT scans showed no significant morphological change, showed a flat p-value distribution between 0 and 1, proving the effectiveness of the algorithm in handling the positioning and DT variations among the different fractions. Both DT and positioning corrections are applied in all the studies performed and presented in the following paragraphs.

The data collected during the trial and the MC simulation samples have been processed with the same reconstruction software and the PCA profiles for each SPB have been obtained. Both the DT and positioning corrections were applied in all cases.

## Results

### Results from the trial data analysis

In this paragraph we present the results of the analysis performed on the first carbon ion treatments of actual patients that have ever been monitored online.

Out of the three patients listed in Table 1, PZA and PZB did not show any evident morphological change: for PZB no re-evaluation CT was planned, as no change was expected; for PZA the re-evaluation CT performed after the first 7 fractions confirmed that no significant change in the morphology occurred. The p-value analysis of PZA and PZB confirmed the consistency of the measured spectra among the different fractions. The obtained distributions are flat, indicating that the measured maps are statistically consistent in all the SPBs and that no significant morphological change occurred in the monitored fractions.

Unlike patients PZA and PZB, PZC showed in the re-evaluation CT a significant difference in the density traversed by the beam. The morphological changes are shown in Fig. 3, where the initial CT used for the treatment planning (CT1) and the re-evaluation one (CT2) are shown respectively on the left and on the right.

**Figure 4:**
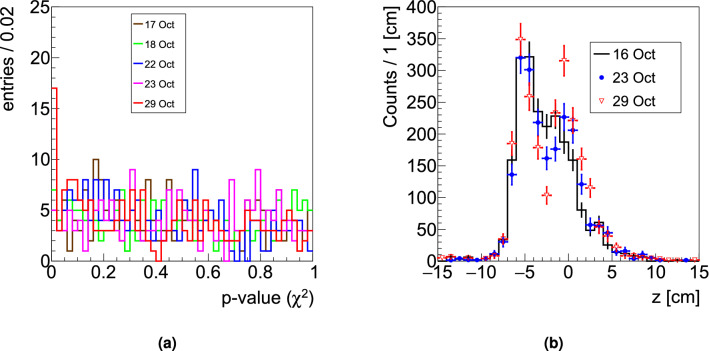
Left: The p-value distribution obtained performing the $\chi ^2$ test between the 1D projections relative to the first measured fraction (16 October) and all the other monitored fractions is shown. Right: Reconstructed fragment emission profiles measured in three different fractions. A clear shape change can be observed due to the nasal cavities emptying.

The results obtained processing the data from the first irradiation configuration (9 fractions) of PZC are presented in Fig. 4. The p-value distribution is shown on the left: a peak at low values arising after the 23rd of October can be observed. The number of SPBs resulting in a p-value < 2% is reported for each monitored fraction in Table 2, demonstrating the impact of a gradual emptying of the nasal cavities occurring after the 23rd of October. In Fig. 4 (right) the modification of the fragment emission profile is observed for a SPB selected requiring a p-value < 2 $\%$.

**Table 2:** Number of SPB for which the $\chi ^2$ test results in a p-value < 2%, for each monitored session.

Fraction date	Fraction number	# of SPB with p-value < 2 %
17 October	2	5 ± 2
18 October	3	7 ± 3
22 October	5	5 ± 2
23 October	6	5 ± 2
29 October	9	17 ± 4

The uncertainty has been evaluated assuming a pure poissonian model.

**Figure 5:**
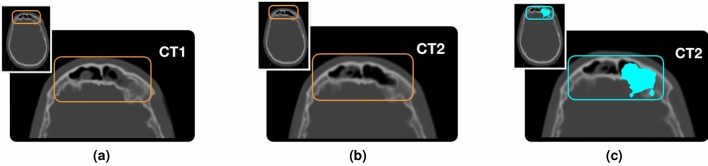
Left: view of CT1 of PZC. Center: view of CT2 of PZC. In both images the region interested by the morphological change, spotted via the re-evaluation CT, is highlighted in orange. The emptying of the region can be clearly seen. Right: CT2 of PZC with superimposed, in cyan, the distribution of the PCAs belonging to the SPBs that have a p-value below 0.02 when testing the consistency between the first monitored fraction and the last one.

The CT information, only for the x,z view, is shown again in Fig. 5 left and center, while the superposition of the patient CT with the image built using the reconstructed PCAs belonging to the SPBs that are marked as discrepant using the p-value criteria (requiring a p-value below 2%) is shown in Fig. 5 right. The PCAs, shown in cyan, are located in the region where the density change occurred.

### MC validation

To cross check the results obtained analysing the data from the clinical trial we ran a full MC simulation in which the CT input, shown in Fig. 3, was taken from PZC, generating the PCAs maps for both fields and both CTs.

**Figure 6:**
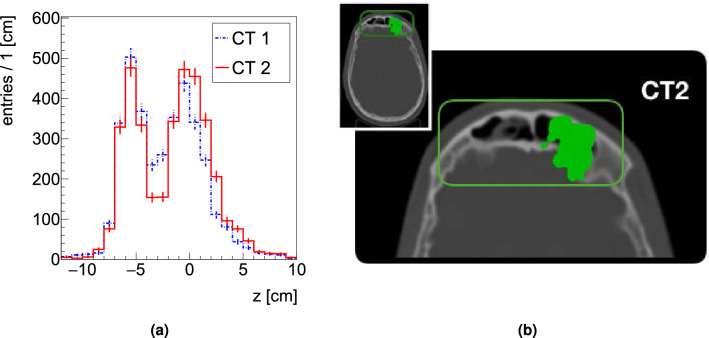
Results of a MC simulation. Left: PCA distributions obtained using respectively CT1 and CT2 as input. The shown SPB has been selected among the ones that have a $\chi ^2$ p-value below 0.02 (for details see text). The results prove that we are observing statistically significant indications of morphological changes. Right: CT2 of PZC with superimposed, in green, the distribution of the PCAs belonging to the SPBs that have a p-value below 0.02 when testing the consistency between CT1 and CT2 (discrepant SPBs).

The p-value distribution from all the SPBs with at least 100 PCAs entries has been obtained and a peak at low p-value has been found, consistently with what expected from the clinical trial results. An example of the distributions obtained for discrepant SPBs (p-value below 0.02) is shown on Fig. 6 (left). While the entrance regions match (the rise of both distributions is found in the same position) the central regions (between − 5 and 0 cm) shows a significantly different behaviour located where the morphological change is expected, matching the clinical trial results shown in Fig. 4 (right).

The correlation of the discrepant SPBs with the morphological change is illustrated in Fig. 6 (right) where the PCAs distribution, selected requiring a p-value below 0.02 and shown in green, is superimposed on the CT consistently with what already done analysing the data and shown in Fig. 5 (right).

### Range monitoring potential

**Figure 7:**
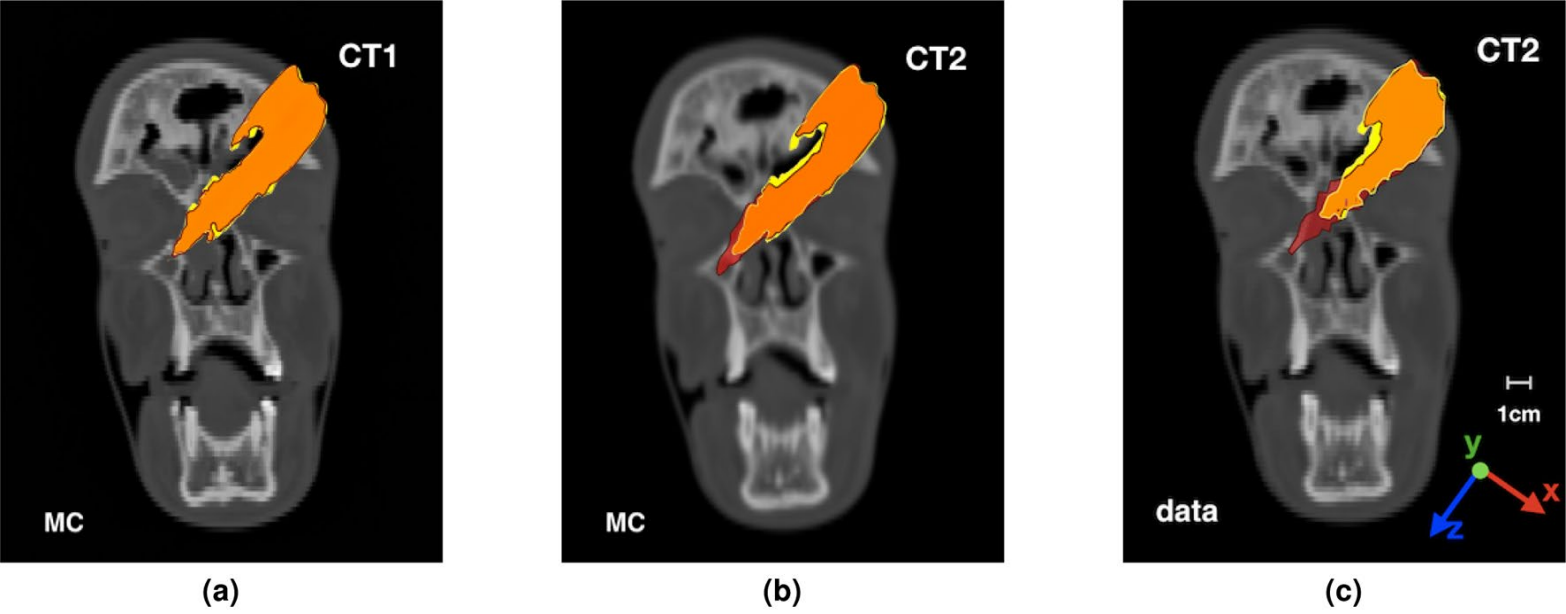
Left: Superposition of the PCA maps obtained using PZC CT1 as input for the MC simulations to generate two independent samples (shown in yellow and red). The orange region identifies the maps overlapping. Center: Superposition of the PCAs maps from the MC samples generated using respectively PZC CT1 (yellow) and CT2 (red) as input. The longer range of the particles in CT2 is highlighted by the red area that exceeds the orange (overlapping) region. Right: PCAs maps obtained from the data collected respectively in the first (yellow) and the last (red) treamtent fractions.

As the range of the primary ions inside the patient depends on the density of the crossed tissues, we have explored the potential of the PCAs map analysis in spotting inter-fraction range variations comparing the maps projections in the horizontal plane, for the MC simulation samples obtained using as input respectively the CT1 and CT2 scans. The result is shown in Fig. 7.

Figure 7a shows the PCA maps agreement when no significant morphological changes are expected. A yellow and a red map have been obtained using two independent samples (same input CT but two different random seeds) and have been superimposed. The orange area is the one in which the yellow and red maps match. While there are some small yellow spots related to the statistical fluctuations in generating the two independent samples, it is clear that the observed maps are consistent.

Figure 7b shows the PCA maps when a significant change is observed. The maps have been obtained using as input the CT1 (yellow) and CT2 (red) scans, and are shown superimposed to the CT2 image. In this case it is clearly visible a region (in red) where the range inside the patient is longer, because of the lower density crossed by the beam. The observed behaviour in this case indicates that the maps analysis could be exploited to detect range variations inside the patient, and used to signal the need of a re-evaluation CT.

Figure 7c shows the same distributions that have, in this case, been built using the data collected in the first fraction (16 October, yellow) and the last one (29 October, red). Despite some, expected, small differences between the MC PCA maps and the measured ones, a remarkable agreement between the data and MC distributions is observed and the range difference can be clearly identified also in this case.

## Discussion

In the present manuscript a technique for spotting morphological changes occurring in patients over the course of carbon ion treatments, based on the measurement of production points of secondary charged fragments originating from the patient, is discussed. The potential of the technique has been studied using the data collected from the treatment of three patients enrolled in the INSIDE clinical trial ongoing at the CNAO facility and cross checked against a dedicated MC simulation based on a detailed description of the CNAO treatment room, of the DP detector, the patient and the corresponding treatment plan.

The DP detector was smoothly operated in the treatment routine of patients providing for the first time information about the charged fragments produced by the beam interaction with the patient tissues and with the RS.

The obtained results demonstrate that the technique is able to spot the insurgence of significant density changes occurring in the first few cm of the patient tissues that are crossed by the beam and that, in these cases, it can suggest the need of an early re-evaluation CT and a treatment re-planning.

The performed analysis, based on the statistical tests done on the 1D projections of the secondary particles production points, gives promising results, although it is not yet optimised and it does not fully exploit the available 3D information. Additional data being collected during the clinical trial will help to define the ultimate sensitivity of the technique in the early spotting of significant morphological changes and to carefully evaluate the impact of the tumor positioning, size and treatment strategy on the achievable sensitivity.

The results presented confirm that the 1D-based analysis approach is robust against the statistical and systematic variations and it does not provide false positives when processing the data from patients in which no significant change occurred. The impact of statistical fluctuations in diluting the technique potential, as well as the actual impact on the clinical work-flow (changes that lead to a treatment re-planning) require more data and a refined (3D) data analysis in order to be conclusive. The clinical trial will end in 2021 with a total of 20 patients, treated with carbon ions, that will be monitored using the DP.
